# A workbook- and audio-administered, telephone-guided, low-intensity cognitive behavioural therapy intervention (EJDeR) for parents of persons treated for cancer during childhood: protocol for the CHANGE-pilot randomised controlled trial

**DOI:** 10.1136/bmjopen-2026-124267

**Published:** 2026-09-22

**Authors:** Joanne Woodford, Christina Reuther, Louise Asberg Dun, Paul Farrand, Kine Johansen, Parent Advisory Board, David A Richards, Filipa Sampaio, Ella Thiblin, Louise von Essen

**Affiliations:** 1 Department of Women's and Children’s Health, Uppsala Universitet, Uppsala, Sweden; 2 Clinical Education Development and Research (CEDAR), University of Exeter, Exeter, UK; 3 Department of Health and Caring Sciences, Western Norway University of Applied Sciences, Bergen, Norway; 4 Department of Public Health and Caring Sciences, Uppsala University, Uppsala, Sweden

**Keywords:** Cancer Survivors, Family, Informal Caregivers, Paediatric oncology, Psychosocial Intervention, MENTAL HEALTH

## Abstract

**Introduction:**

Childhood cancer and its treatment place substantial demands on parents, causing significant disruptions in multiple areas of life. Parents report psychological, psychosocial, and economic consequences related to their child’s illness. In Sweden, psychosocial support is available to parents via paediatric oncology care during cancer treatment; however, only a minority of parents have access to psychological support after treatment completion. Increasing and improving access to such support could enhance parental mental well-being and help prevent or alleviate negative consequences. One potential solution to provide support is the EJDeR intervention, a co-created workbook- and audio-administered, telephone-guided, low-intensity cognitive behavioural therapy (LICBT) intervention for parents of persons treated for cancer during childhood. Objectives are to examine the feasibility of (1) EJDeR delivered via workbooks, audio files and video case vignettes with telephone guidance from a parent guide, and (2) planned study procedures for a future superiority randomised controlled trial of EJDeR.

**Methods and analysis:**

This is a parallel-group, two-arm, pilot randomised controlled trial with a 1:1 allocation ratio. A Parent Advisory Board (PAB) contributes to the research. We aim to recruit 60 parents of persons diagnosed with cancer at age 0–18 years whose treatment was completed 3 months to 5 years earlier. Participants will be randomly allocated to EJDeR plus usual care or usual care alone. EJDeR is delivered over 12 weeks via workbooks, audio files and video case vignettes, with telephone guidance from a parent guide. We will examine feasibility outcomes related to procedural, intervention, and methodological uncertainties using a priori progression criteria following a GO-AMEND-STOP progression framework. We will measure clinical (mental well-being, symptoms of depression, anxiety, and post-traumatic stress) and health economic (health-related quality of life and societal resource use) outcomes at baseline, post-treatment (12 weeks), and follow-up (6 months post-treatment). Data analysis is primarily descriptive.

**Ethics and dissemination:**

This trial was approved by the Swedish Ethical Review Authority (Dnr 2025–09024-01, approval date: 2 February 2026). We will obtain written informed consent from all participants and disseminate findings in collaboration with the PAB through peer-reviewed publications, conference presentations, and non-academic dissemination activities such as co-writing plain language summaries and dissemination via childhood cancer organisations.

**Trial registration number:**

ISRCTN51549508.

**STRENGTHS AND LIMITATIONS OF THIS STUDY**

- This pilot randomised controlled trial uses a priori progression criteria following a GO-AMEND-STOP progression framework to systematically assess procedural, intervention, and methodological uncertainties for a future superiority randomised controlled trial.
- The intervention was co-created with parents of persons treated for cancer during childhood with modifications informed by previous feasibility trial findings, addressing identified barriers to engagement.
- A Parent Advisory Board will contribute throughout the trial, for example, to study design, conduct, analysis, interpretation, and dissemination.
- Research team members conducting assessments will not be blinded to group allocation, and therefore, we cannot examine the feasibility of blinding for a future evaluation of EJDeR in a superiority randomised controlled trial.
- The study is limited to Swedish-speaking parents living in Sweden, which limits generalisability to other settings and populations. Future research should include cultural adaptation of the intervention to improve its relevance for linguistically and culturally diverse parents.

## Introduction

 Cancer is a leading cause of death in children; however, advances in treatment have increased survival rates to approximately 80% in high-income countries.^1^ A childhood cancer diagnosis causes significant and lasting disruption to parents’ lives. Parents report fatigue, fear of recurrence, loneliness, low mood, relationship challenges, uncertainty about long-term prognosis and potential late effects, worry,^2–6^ and difficulties navigating life after treatment completion.^7^ Subgroups report symptoms of depression (14%),^8^ anxiety (20%),^8^ post-traumatic stress (PTSS) (21–44%),^9^ and health-related quality of life clinically worse than perfect health (39%).^10^ Symptoms of depression, anxiety, and PTSS among parents correlate with children’s global distress,^11^ and there is a relationship between parents’ psychosocial adjustment and children’s quality of life.^12^ Childhood cancer also negatively affects parents’ employment and earnings.^13–15^ Parents have described the transition from hospital care to the time after cancer treatment as particularly challenging, as healthcare is abruptly withdrawn, with some parents reporting being inadequately prepared for the post-treatment period.^2^ While psychosocial support is available to parents via paediatric oncology care during cancer treatment,^16^ only a minority of parents in Sweden receive psychological support after treatment completion, and access declines over time.^17^ Addressing parents’ support needs may enhance mental well-being and prevent or alleviate symptoms of emotional distress^11^ as well as broader concerns, for example, loneliness, parenting difficulties, relationship challenges and sleep difficulties.^5^

### The internet-administered guided EJDeR intervention

**Figure 1:**
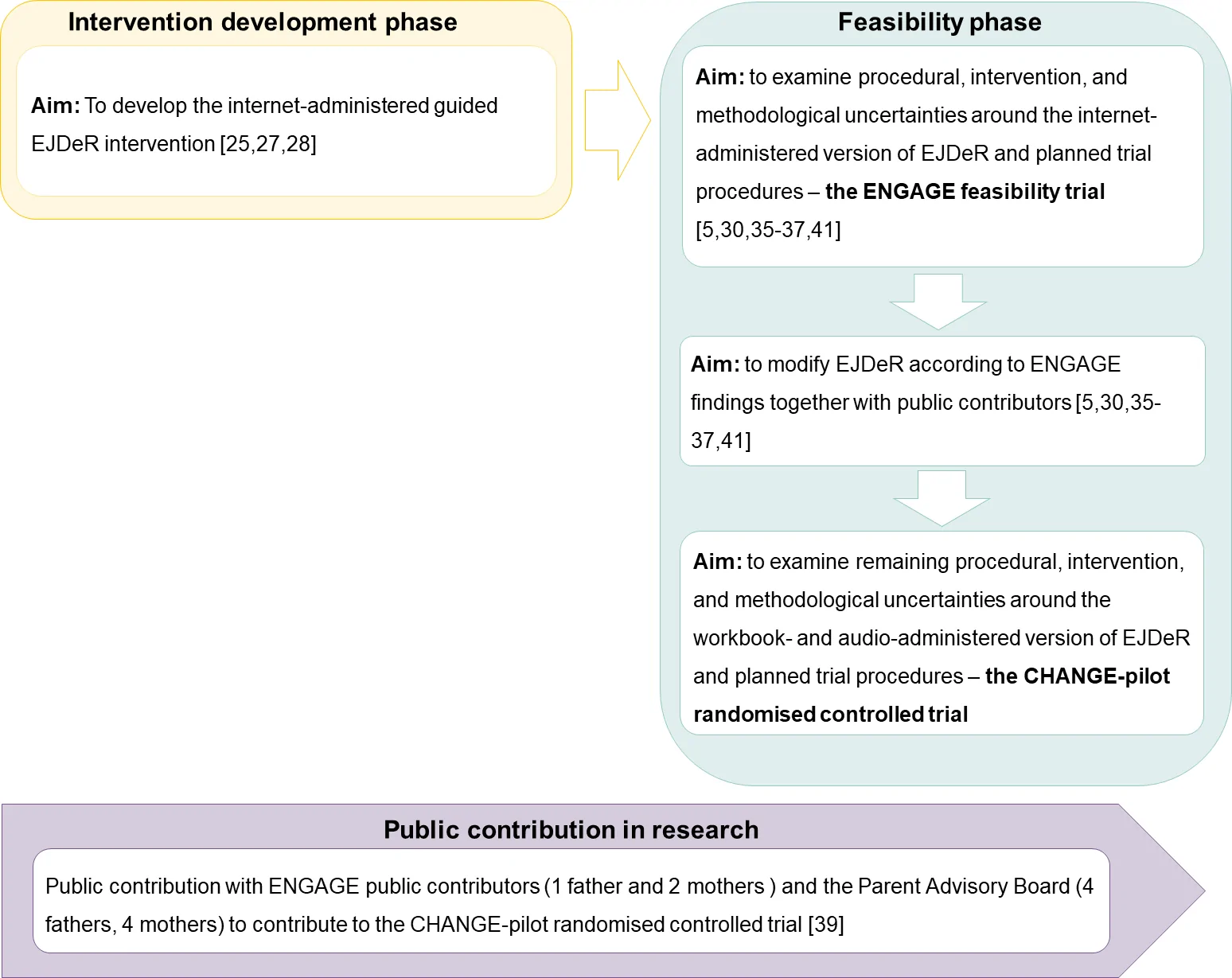
Intervention development and feasibility testing.

Given the aforementioned unmet support needs of parents of persons treated for cancer during childhood, we conducted iterative intervention development and feasibility research (figure 1), following the Medical Research Council (MRC) framework.^18^

Internet-administered cognitive behavioural therapy (iCBT) can reduce symptoms of depression and anxiety^19^ as well as promote mental well-being,^20^ and parents’ unmet need of psychological support could be met through low-intensity cognitive behavioural therapy (LICBT). LICBT interventions can be delivered through a range of cognitive behavioural therapy self-help interventions, for example, via audio,^21^ internet, smartphone applications, and/or workbooks.^22^ There is evidence for larger effect sizes for LICBT interventions guided by a trained professional versus self-administered.^23^ In our previous research, we found that 74% of parents of persons treated for cancer during childhood would accept, or maybe accept, iCBT if offered.^24^ Our cognitive behavioural conceptualisation of distress in the population identified symptoms of depression and symptoms associated with generalised anxiety disorder, for example, persistent future-orientated anxiety and worry, as core difficulties experienced by parents.^25^ Given interventions for caregivers are more likely to demonstrate a beneficial effect when designed for the target population,^26^ as the first step, we co-created the internet-administered LICBT intervention EJDeR,^27 28^ delivered via the U-CARE portal,^29^ with guidance provided by e-therapists via telephone or video-conferencing (according to preference) and written messages online.

### The ENGAGE feasibility trial

Research from the intervention development phase informed the feasibility phase (figure 1) where we conducted the single-arm feasibility trial ENGAGE^30^ to examine procedural, intervention, and methodological uncertainties around the internet-administered version of EJDeR and planned trial procedures for a future evaluation of EJDeR in a superiority randomised controlled trial (RCT). Progression criteria were set a priori, following a GO-AMEND-STOP progression framework.^31^ We enrolled 75 participants (56 identified via the Swedish Childhood Cancer Registry and Swedish Tax Agency’s Population Registry, and a further 19 via other recruitment strategies), of which 72 completed baseline assessments and gained access to EJDeR.

Five progression criteria met GO: (1) 11.0% (56/509) were enrolled of the total potential participant population invited via the Swedish Childhood Cancer Registry and Swedish Tax Agency’s Population Registry, criteria ≥9.0%; (2) 24% (18/75) dropped out of the trial and 23.6% (17/72) dropped out of the intervention, criteria ≤30.0%; (3) 0.01–3.9% of questionnaire items were missing, criteria ≤10.0%; (4) 98.7% (74/75), 84.4% (54/64) and 81.4% (48/59) completed telephone interviews at baseline, post-treatment (12 weeks), and follow-up (6 months post-treatment), respectively, of those remaining in the study at each time point, criteria ≥70.0%; and (5) no substantial negative consequences were reported. Two criteria met AMEND: (1) 65.6% (42/64) and 67.8% (40/59) completed online assessments at post-treatment (12 weeks) and follow-up (6 months post-treatment) respectively of those remaining in the study at each time-point, criterion ≥70.0%); and (2) 47.9% (34/71) completed the minimum treatment dose completion, criterion ≥50.0%.

In ENGAGE, clinical outcomes included symptoms of depression^32^ and anxiety.^33^ However, only 21.0% reported a clinically significant level of symptoms of depression and 22.0% symptoms of anxiety at baseline, indicating these symptoms may not be the most appropriate primary outcomes in a future superiority RCT. In ENGAGE, before working with EJDeR, parents reported non-specific symptoms of mental distress, including lack of energy and motivation, sleep difficulties and stress, as well as wider difficulties with parenting, productivity and relationships.^5^ Globally, there is a growing focus on promoting mental well-being to protect from poor mental health and mental health disorders.^34^ Promoting mental well-being might be especially suitable for parents of persons treated for cancer during childhood, and mental well-being may be an appropriate primary outcome in a future evaluation of EJDeR.

Process evaluation findings showed that parents^35^ and e-therapists^36^ found EJDeR acceptable and relevant. Parents reported that EJDeR provided structure but wanted more content tailored to their experience of being a parent of a person treated for cancer during childhood. Additionally, they preferred fewer online assessments, as well as improved smartphone and tablet compatibility.^35^ E-therapists^36^ reported challenges supporting trauma-related difficulties and difficulties navigating the U-CARE portal.^29^ Both groups reported favouring real-time person-to-person communication (telephone or video-conferencing) to personalise support and strengthen the therapeutic relationship.^35 36^ Parents who started with the LICBT technique worry management were less likely than those starting with the LICBT technique behavioural activation^37^ to complete the minimum treatment dose suggesting module sequencing affects engagement.^38^

### The CHANGE-pilot RCT

ENGAGE findings indicated EJDeR and study procedures overall were acceptable and feasible.^30^ However, two progression criteria, relating to completing the minimum treatment dose and data completeness, did not meet the GO criterion. Therefore, we progress via an external pilot RCT with progression criteria set^31^ (the CHANGE-pilot RCT, figure 1) to examine remaining procedural, intervention, and methodological uncertainties around the workbook- and audio-administered version of EJDeR and planned trial procedures. Findings will inform the design and conduct of a future full-scale evaluation of EJDeR in a superiority RCT of EJDeR plus usual care (intervention group) versus usual care alone (control group).

#### Public contribution

We recruited a Parent Advisory Board (PAB; four fathers, four mothers of persons treated for cancer during childhood) to contribute to the CHANGE-pilot RCT.^39^ We held online workshops with the PAB coordinated by one research team member (internal coordinator) and one father of a person treated for cancer during childhood (external coordinator). Coordinators recorded PAB suggestions in an impact log.^40^ Suggestions informed recruitment and retention procedures, for example, compensation for completing data collection; data collection procedures, for example, book upcoming interviews via SMS; outcomes and questionnaires, for example, measure positive outcomes such as mental well-being and reduce the number of questionnaires; and EJDeR delivery mode, for example, audio-administration. PAB contribution continues throughout the CHANGE-pilot RCT.^39^

#### Modifications to EJDeR content, delivery and guidance

We modified EJDeR content according to ENGAGE findings^5 30 35–37 41^ together with ENGAGE public contributors (one father and two mothers of persons treated for cancer during childhood) and the PAB.^39^ Main modifications were to (1) include additional cancer-related examples in self-help exercises; (2) add video case vignettes focused on parents’ recovery stories; (3) provide psychoeducation on motivation; (4) enhance existing motivation self-help exercises; (5) add an additional psychoeducation and resources module on common reactions to traumatic events, parenting, relationships and sleep, as well as providing information about relevant national resources available for parents of persons treated for cancer during childhood; and (6) revise language to emphasise mental well-being (see online supplemental table S1).

Both parents^35^ and e-therapists^36^ found the internet-administered delivery of EJDeR complex and voiced a preference for person-to-person communication. We conducted ENGAGE during the COVID-19 pandemic, with some participants reporting being tired of spending time in front of a computer.^35^ Research suggests digitalisation is associated with ‘digital fatigue’, that is, feeling physically and mentally exhausted,^42^ and excessive screen time negatively affects mental health.^43^ The PAB suggested providing EJDeR in audio form could enhance engagement by allowing parents to listen to the intervention texts without needing to use a screen, thereby improving accessibility. While research on audio-administered mental health interventions is limited, there is some evidence for improving insomnia among breast cancer survivors,^44^ depression in older adults^45^ and stabilising refugees with post-traumatic stress disorder,^46^ indicating that an audio-administered intervention may be a promising solution for delivering psychological support.

A core element of LICBT interventions associated with effectiveness^47^ is engagement with self-help exercises.^21^ We therefore developed a workbook- and audio-administered, telephone-guided LICBT intervention version of EJDeR. Workbooks contain intervention text, psychoeducation, illustrations, written case vignettes, and self-help exercises, and are designed to facilitate engagement with intervention content and self-help exercises. Providing both a workbook and audio-administered intervention may facilitate matching delivery modality to preferred learning style (ie, listening or reading, depending on preference).^21^ Guidance over the telephone from a parent guide (referred to as e-therapists in ENGAGE) is included; given telephone-guided psychological interventions have been found to be as effective as face-to-face interventions for depression^48^ and do not compromise the therapeutic relationship.^49^

### Rationale for the CHANGE-pilot RCT

#### Procedural uncertainties

ENGAGE was a single-arm feasibility trial.^30^ In the CHANGE-pilot RCT, we will examine the feasibility of randomisation procedures. We have amended data collection procedures from those used in ENGAGE, for example, we will provide participants with a gift voucher (100 SEK, ≈ €9) after a completed assessment at post-treatment (12 weeks) and follow-up (6 months post-treatment), respectively, and collect data via telephone rather than online. We have also reduced the number of self-report questionnaires. We will examine procedural uncertainties, for example, recruitment, retention and data collection rates, to assess whether we can recruit and retain participants and collect data with low rates of missingness in a RCT with amended data collection procedures from those used in ENGAGE.

#### Intervention uncertainties

Since conducting ENGAGE, we modified EJDeR with respect to content, mode of delivery, and type of guidance and defined a revised minimum treatment dose (see Minimum treatment dose). Uncertainties exist regarding the proportion of participants who will complete the minimum treatment dose when receiving the modified intervention, and regarding parent guide fidelity to the EJDeR guidance protocols. We will therefore examine these intervention uncertainties to ensure the intervention is feasible to participants and parent guides.

#### Methodological uncertainties

In ENGAGE, we did not specify a primary clinical outcome measure(s) for a future superiority RCT, nor did we measure mental well-being. We will measure mental well-being as a proposed primary clinical outcome for a future superiority RCT, but sensitivity to change in the population is unknown. The variance and estimated between-group differences in follow-up scores for mental well-being and correlation with baseline scores are unknown. We will require this information to inform a sample size calculation for a future superiority RCT.^50^

### Objectives

Objectives are to examine the feasibility of (1) EJDeR delivered via workbooks, audio files, and video case vignettes with telephone guidance from a parent guide and (2) planned study procedures for a future superiority RCT of EJDeR.

We will examine procedural, intervention and methodological uncertainties via the following research questions:

#### Procedural uncertainties

1. Is there a difference in sociodemographic characteristics between those invited versus those who consent?

2. What proportion of those invited consent, are assessed for inclusion, are included, and are randomised?

3. What proportion of the intervention and control groups complete questionnaires at baseline, post-treatment (12 weeks), and follow-up (6 months post-treatment)?

4. What proportion of responses are missing in each questionnaire for the intervention and control group at baseline, post-treatment (12 weeks), and follow-up (6 months post-treatment)?

#### Intervention uncertainties

5. What proportion of the intervention group completes the minimum treatment dose?

#### Methodological uncertainties

6. What is the variance in mental well-being for the intervention and control group at post-treatment (12 weeks) and follow-up (6 months post-treatment), and is there a correlation with mental well-being at baseline?

7. Is there a difference in mental well-being between the intervention and control group at post-treatment (12 weeks) and follow-up (6 months post-treatment)?

## Methods and analysis

### Public contribution

Suggestions made by the PAB and implemented in the CHANGE-pilot RCT protocol are summarised in table 1. PAB contribution will continue throughout the trial, including approximately four online workshops to contribute to analysis and interpretation of findings and to inform dissemination outside of academia, for example, local dissemination strategies, and plain language summaries.^39^ Two PAB members will attend trial steering committee (TSC) meetings to discuss trial progress and problem-solve challenges and will provide updates to other PAB members. While PAB members cannot participate in the trial, those interested are provided access to the EJDeR intervention but without guidance from a parent guide.

**Table 1:** Suggestions made by the Parent Advisory Board and implemented in the protocol for CHANGE-pilot RCT

Category	Suggestion
Recruitment and retention procedures	Send SMS before making a recruitment reminder call.
	Provide participants with monetary compensation.
Data collection procedures	Book upcoming interviews via SMS.
	Reschedule interviews via SMS.
Outcomes and outcome measures	Ask about potential participants’ country of birth in the inclusion interview.
	Ask about potential participants’ parents’ country/countries of birth in the inclusion interview.
	Ask about potential participants’ native language in the inclusion interview.
	Reduce the number of outcome measures focused on specific symptoms of depression and anxiety, for example, executive difficulties and intolerance of uncertainty, and measure positive outcomes such as mental well-being.
	Ensure research team members collecting data have some basic knowledge about childhood cancer, for example, diagnoses and treatments.
EJDeR delivery	Administer EJDeR in audio form.
	Ensure parent guides have some basic knowledge about childhood cancer, for example, diagnoses and treatments.

SMS, short message service.

### Study design

**Figure 2:**
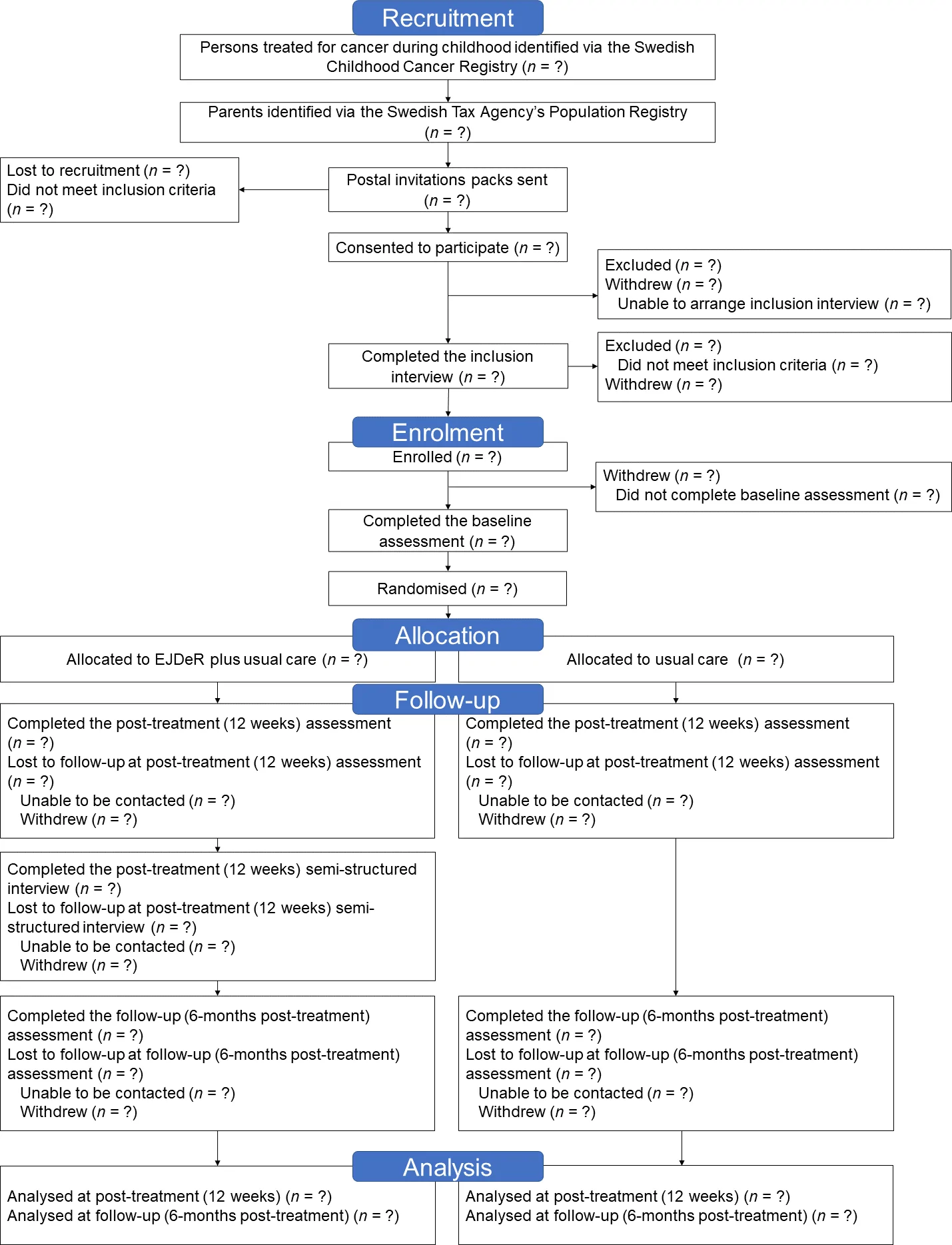
Consolidated Standards of Reporting Trials (CONSORT) diagram.

This is a parallel-group, two-arm, pilot RCT with a 1:1 allocation ratio. The protocol follows the CONSORT 2010 extension to randomised pilot and feasibility trials^51^ and the Standard Protocol Items: Recommendations for Interventional Trials (SPIRIT) 2025 checklist.^52^ A CONSORT flow diagram is shown in figure 2. We undertake a mixed-methods process evaluation embedded within the trial, with methods described in a separate protocol paper.^53^

#### Protocol changes

This protocol is published after trial commencement (start date: June 2026). If trial protocol amendments are required, approval will be sought from the Swedish Ethical Review Authority, and amendments will be communicated to the trial registry (ISRCTN), TSC, and participants if applicable.

### Trial setting

We coordinate the trial from Uppsala University, Sweden. Due to EJDeR being a workbook- and audio-administered intervention, participants can engage with the intervention when and where they prefer.

### Eligibility criteria

Participants must (1) be a parent of a person diagnosed with cancer at age 0–18 years whose treatment was completed 3 months to 5 years earlier, (2) understand, speak, read and write Swedish well enough to access the study material, provide informed consent, answer questionnaires, participate in interviews and work with EJDeR, and (3) have a mobile phone with a telephone number.

### Intervention and control groups

We will randomly assign participants to EJDeR plus usual care (intervention group) or usual care alone (control group). We chose usual care as the comparator because this reflects current standard care in Sweden where only a minority of parents of persons treated for cancer during childhood receive psychological support after treatment completion; it allows us to assess the feasibility of delivering EJDeR in addition to usual care, and it informs the design of a future superiority RCT comparing EJDeR plus usual care versus usual care alone. Procedures for identifying and responding to participant deterioration or disclosure of risk during the trial are described under Harms.

#### EJDeR plus usual care (intervention group)

EJDeR is delivered over 12 weeks and includes five modules, separated into five workbooks: (1) Introduction and Psychoeducation; (2) Behavioural Activation (for symptoms of depression/low mood); (3) Worry Management (for symptoms of anxiety); (4) Relapse Prevention; and (5) Additional Psychoeducation and Resources. Workbooks include text, self-help exercises, illustrations, and written case vignettes. Audio recordings of workbook text, including self-help exercise instructions, and video case vignettes are provided via an online platform. Parents are encouraged to work with only one LICBT technique at a time and may use the same technique for several weeks.

The Introduction and Psychoeducation Module includes information on mental well-being in the context of being a parent of a person treated for cancer during childhood, an introduction to the CBT model and the LICBT techniques (behavioural activation and worry management), goal setting, and motivation. The Behavioural Activation Module targets behavioural avoidance, which is common in depression.^54^ The Worry Management Module includes two LICBT techniques: worry time^55^ and problem-solving,^56^ and targets emotional and behavioural avoidance, unhelpful coping strategies,^41^ and intolerance of uncertainty,^57^ all common in anxiety. The Relapse Prevention Module supports the parent to summarise their work with EJDeR and plan how to continue using the LICBT technique(s) in the future. The Additional Psychoeducation and Resources Module includes psychoeducation on motivation, common reactions to traumatic events, imagination self-help exercises to help with emotion regulation and psychoeducation on parenting, relationships and sleep, as well as information about relevant national resources available for parents of persons treated for cancer during childhood, for example, family support and financial guidance.

##### Parent guides

EJDeR is guided by a parent guide with documented advanced CBT skills. Parent guides attend a training workshop on (1) LICBT, (2) psychological and psychosocial concerns experienced by parents of persons treated for cancer during childhood, (3) basic knowledge about childhood cancer, for example, diagnoses and treatments, (4) the structure of EJDeR, and (5) telephone guidance protocols.^58^ A licensed psychologist with experience of the population and the external PAB coordinator with lived experience being a parent to a person treated for cancer during childhood deliver the training workshop. A licensed clinical psychologist or clinical expert in LICBT provides parent guides with weekly supervision (case management and clinical skills development),^59^ with additional at-need supervision provided if needed.

##### Telephone guidance

Guidance follows structured LICBT protocols.^60 61^ During the first week of the intervention, the parent guide schedules an initial assessment session (≈45 min) with the parent. The parent guide assesses the participant’s main psychological difficulties and provides the rationale (ie, how the techniques work) for behavioural activation and worry management. Working collaboratively with the participant they agree which LICBT technique to start working with. Participants work through the Introduction and Psychoeducation Module and begin working with the chosen LICBT technique, for example, by reading the workbook, listening to audio recordings of workbook text, and completing exercises. Thereafter, the parent guide provides up to 10 weekly telephone ‘check-in’ sessions (≈15 min) to the participant to review progress, problem-solve any difficulties experienced, and plan next steps. A relapse prevention session (≈30 min) is provided during week 12.

Sessions will be audio recorded (with consent) and we will select a random 15% of recorded sessions to examine parent guide fidelity to the EJDeR guidance protocols. The sampling strategy and fidelity assessment method are described in full in the process evaluation protocol.^53^ Guidance sessions can still take place if the participant does not consent to them being recorded. Parent guides will record data on intervention implementation and self-reported data from participants on intervention engagement in a session log and keep clinical notes to facilitate guidance.

**Figure 3:**
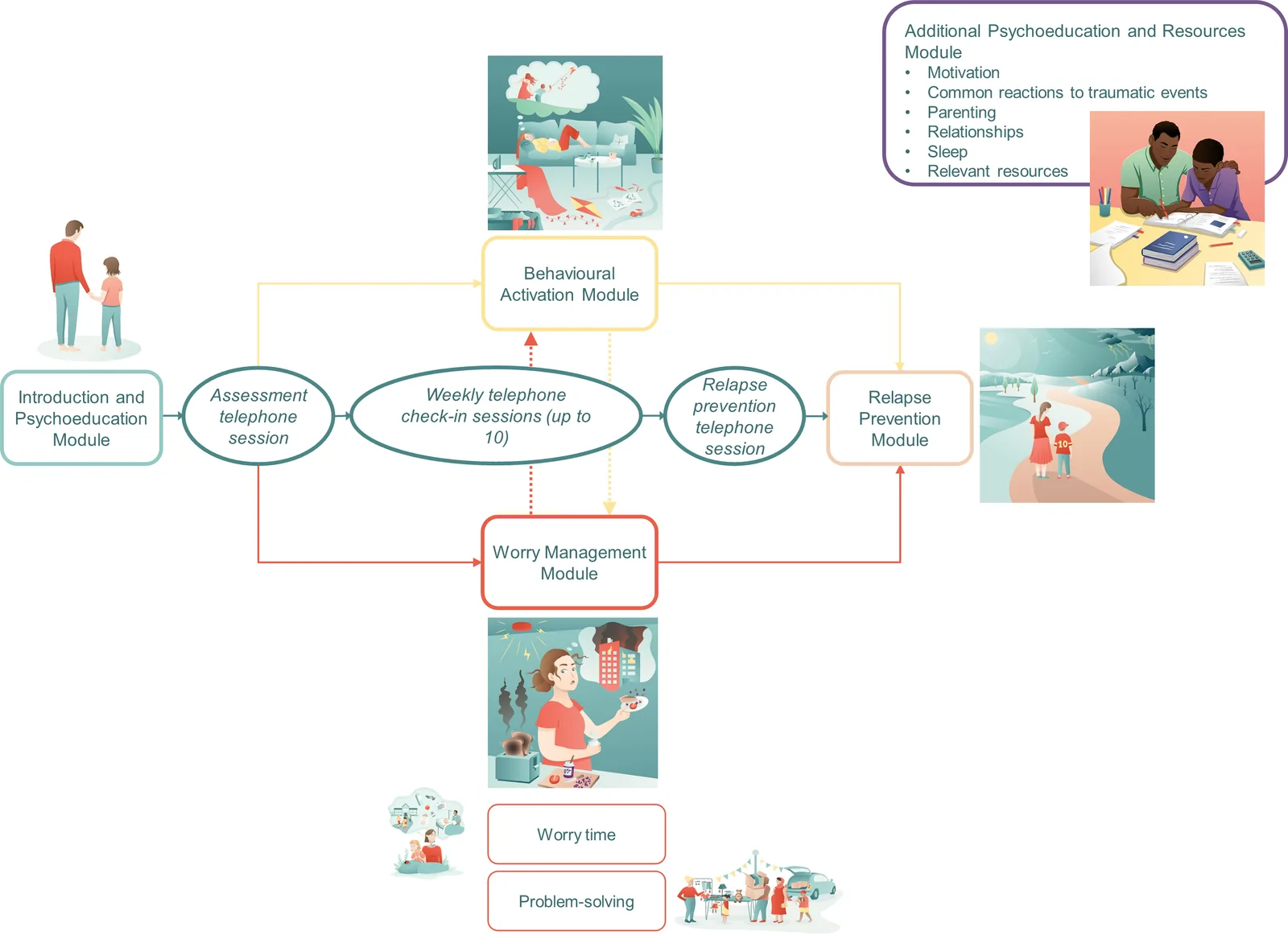
Structure of the EJDeR intervention.

An overview of the EJDeR intervention structure is provided in figure 3. Participants randomly assigned to EJDeR will be able to access usual care.

##### Minimum treatment dose

The minimum treatment dose is defined as participants attending the initial assessment session with the parent guide and completing at least 4 weekly telephone guidance sessions with the parent guide. This is based on evidence suggesting a minimum of four LICBT guidance sessions are required to achieve an intervention effect.^62^

### Usual care

Participants can continue with usual care,^63^ which may include any type of care, for example, psychological support in accordance with national guidelines for treatment of depression and anxiety,^64^ and/or prescribed psychotropic drugs via primary care. Participants allocated to the control group will not be offered EJDeR at the end of the trial.

### Withdrawal

Without giving a reason, participants can contact the research team to withdraw from the trial and/or the intervention by contacting the research team via telephone or SMS or during a conversation with their parent guide. Participants in the intervention group may withdraw from the intervention but continue trial participation, for example, answer questionnaires and vice versa.

### Outcomes

#### Primary feasibility outcomes

Feasibility outcomes relate to procedural, intervention and methodological uncertainties following recommendations for designing and conducting pilot RCTs.^31 51^ We will follow a ‘GO-AMEND-STOP’ progression framework, with STOP indicating ‘do not progress to a future superiority RCT’, AMEND indicating ‘modifications needed to progress to a future superiority RCT’, and GO indicating ‘progress to a future superiority RCT’.^31^ Table 2 summarises feasibility outcomes and progression criteria indicating GO.

**Table 2:** Feasibility outcomes and progression criteria indicating GO

Feasibility outcome	Progression criteria indicating GO	Rationale
Procedural uncertainties		
Proportion invited randomised	≥9.0%	Informed by the enrolment rate (11.0%) found in ENGAGE, with the percentage lowered to account for randomisation potentially leading to lower enrolment rates
Proportion completing all questionnaires at baseline, post-treatment (12 weeks), and follow-up (6 months post-treatment)	≥70.0%	Informed by progression criteria set for ENGAGE
Proportion of responses missing in each questionnaire at baseline, post-treatment (12 weeks), and follow-up (6 months post-treatment)	≤10.0%	Informed by research suggesting missing data ≥10.0% is considered substantial and statistical analysis is likely to be biased^76^
Intervention uncertainties		
Proportion completing the minimum treatment dose	≥65.0%	Informed by research suggesting an average 64.0% of participants adhere to guided web-based mental health interventions^77^

#### Clinical and health economic outcomes

We will collect clinical and health economic outcomes at baseline, post-treatment (12 weeks), and follow-up (6 months post-treatment) via telephone.

##### Primary clinical outcome

Mental well-being is measured with the Questionnaire on Well-Being (QWB).^65^ The QWB contains 18 items measuring subjective well-being and psychological functioning. It has excellent reliability, with Cronbach’s alpha between 0.93 and 0.94, and high test-retest reliability.^65^ Scores range from 0 to 72, with a higher score indicating better well-being.

##### Secondary clinical outcomes

Symptoms of depression are measured with the Patient Health Questionnaire 8-item scale (PHQ-8).^66^ Scores range from 0 to 24, with a higher score indicating higher depression severity.

Symptoms of anxiety are measured with the Generalised Anxiety Disorder 7-item scale (GAD-7).^33^ Scores range from 0 to 21, with a higher score indicating higher anxiety severity.

Post-traumatic stress symptoms are measured with the Post-traumatic Stress Disorder Checklist-Civilian version (PCL-C).^67^ Scores range from 17 to 85, with a higher score indicating higher PTSS severity.

##### Health economic outcomes

Health-related quality of life is measured with the 10-item Recovering Quality of Life (ReQoL-10).^68^ The ReQoL-10 contains 10 items assessing mental health-related quality of life and one item assessing physical health. Scores range from 0 to 44, with a higher score indicating better quality of life.

Societal resource use is measured with a Resource Use Questionnaire (RUQ),^69 70^ adapted by our research team in collaboration with the PAB. The RUQ includes questions from the Trimbos and Institute of Medical Technology Assessment Cost Questionnaire for Psychiatry^69^ and PECUNIA Resource Use Measurement Instrument.^70^ It covers healthcare utilisation (primary care visits, specialist visits, emergency department visits and other healthcare services), medication use, support groups, helplines, and absenteeism and presenteeism related to both paid and unpaid work, and personal expenses.

We will use a micro-costing approach to estimate the costs associated with delivering EJDeR, including time for initial assessment and weekly telephone sessions with parent guides, supervision, training, equipment, developing and producing workbooks, producing audio files, printing, distribution, and administration.

### Process outcomes

We will collect process outcomes at post-treatment (12 weeks) for the intervention group. Experience of the content and delivery of EJDeR is measured with the Theoretical Framework of Acceptability Questionnaire (TFAQ).^71^

Satisfaction with the relationship with the parent guide is measured with the Working Alliance Inventory – Short Revised (WAI-SR).^72 73^

We will conduct semi-structured interviews, described in a separate protocol paper for the embedded process evaluation.^53^

### Harms

We define harms as serious adverse events (SAEs), that is, any expected or unexpected adverse event, related, possibly related or unrelated to the EJDeR intervention or the trial, that results in death, a life-threatening adverse event or requires hospitalisation, which includes suicide attempts and self-harm. All SAEs are reported to the principal investigator (LvE).

We will assess SAEs through spontaneous reporting by participants to research team members or parent guides and ongoing monitoring by parent guides during intervention delivery.

A licensed psychologist will train research team members working with recruitment and data collection to identify signs of serious mental illness and/or risk of suicide. In acute situations, for example, where a participant discloses an immediate risk to their safety, research team members will contact emergency services regardless of the participant’s response. In non-acute situations, for example, suicidal ideation without immediate risk, we will inform the participant that we would like a licensed psychologist in our research team to contact them, and, if the parent gives permission, the licensed psychologist will contact them within two working days to conduct a risk assessment. If the participant does not give permission, they are encouraged to contact primary care. If a parent is assessed as needing more support than we can provide within the trial, we will direct them to appropriate mental health services and exclude them from the trial. If a parent guide identifies signs of serious mental illness (including deterioration) and/or risk of suicide, for example, when receiving telephone guidance from the parent guide, the parent guide will conduct the risk assessment immediately and consult with the parent guide supervisor if needed.

### Trial timeline

An overview on the trial timeline, that is, the time schedule of pre-enrolment, enrolment and post-randomisation activities is provided in table 3.

**Table 3:** Overview of the trial timeline: pre-enrolment, enrolment and post-randomisation

Timepoint	Pre-enrolment	Enrolment	Post-randomisation		
			Intervention	Post-treatment (12 weeks)	Follow-up (6 months post-treatment)
Identify persons treated for cancer during childhood via the Swedish Childhood Cancer Registry	X				
Identify parents of persons treated for cancer during childhood via the Swedish Tax Agency’s Population Registry	X				
Informed consent	X				
Inclusion interview		X			
Baseline assessment		X			
Randomisation		X			
EJDeR plus usual care			X		
Usual care			X		
**Assessments**					
*Clinical outcomes*					
QWB		X		X	X
PHQ-8		X		X	X
GAD-7		X		X	X
PCL-C		X		X	X
*Health economic outcomes*					
ReQoL-10		X		X	X
RUQ		X		X	X
*Process outcomes*					
TFAQ				X*	
WAI-SR				X*	
Semi-structured interview				X†	

* Intervention group only.

† Semi-structured interviews are described in a separate process evaluation protocol.

GAD-7, Generalised Anxiety Disorder 7-item scale; PCL-C, Post-traumatic Stress Disorder Checklist-Civilian version; PHQ-8, Patient Health Questionnaire 8-item scale; QWB, Questionnaire on Well-Being; ReQoL-10, 10-item Recovering Quality of Life; RUQ, Resource Use Questionnaire ; TFAQ, Theoretical Framework of Acceptability Questionnaire; WAI-SR, Working Alliance Inventory – Short Revised .

### Sample size

We will recruit 60 participants (30 to the intervention group, 30 to the control group), consistent with the median sample size per arm reported for pilot trials.^74^ With 30 participants per group, we can estimate proportions, for example, completion rates, with reasonable precision: 9.0% randomisation rate (approximately 60/667 invited; 95% CI 7.0% to 11.4%), 70.0% completion of all questionnaires at baseline, post-treatment (12 weeks), and follow-up (6 months post-treatment) (42/60; 95% CI 57.4% to 80.2%), and completion of the minimum treatment dose in at least 65% of participants in the intervention group (20/30; 95% CI 46.0% to 83.6%). These estimates will provide adequate information to inform progression decisions.

### Recruitment

#### Identification and invitation

Personal identification numbers of persons diagnosed with cancer at age 0–18 years whose treatment was completed 3 months to 5 years earlier are identified via the Swedish Childhood Cancer Registry (National Quality Registry, initiated in 1982). Their parents, with addresses, are subsequently identified via the Swedish Tax Agency’s Population Registry using their online secure file transfer system (e-transport), and the transfers are managed by Uppsala University IT Services.

We will randomise parents identified via the Swedish Tax Agency’s Population Registry to recruitment blocks of approximately 100 parents using computer-generated simple randomisation, with one recruitment block of parents invited per week until the sample size of 60 participants is reached or a recruitment period of 3 months has passed. To minimise the risk of inviting a parent of a person who has died since receiving data from the Swedish Childhood Cancer Registry, we will request up-to-date information concerning their living status from the Swedish Tax Agency’s Population Registry via Uppsala University IT Services on the same day as sending invitation packs to their parent via post.

We will send invitation packs via post including an invitation postcard, an invitation letter, a participant information sheet, two copies of the consent form (one copy can be kept) and a freepost return envelope. Invitation letters are personalised with the parent’s name, as evidence suggests personalisation can improve recruitment rates.^75^ Those who wish to participate provide consent by returning the consent form in the freepost return envelope. Potential participants who want more information can contact the research team via telephone or SMS.

#### Reminders for those who have not consented within three weeks

If an invited potential participant has not consented within 3 weeks of sending the invitation, we will attempt to identify their telephone number online via internet search engines providing public access to Swedish citizens’ telephone numbers. If a telephone number is identified, we will send an SMS informing that we will make up to three attempts to reach them via telephone over the next 2 weeks. The number of reminder attempts is informed by ENGAGE findings, where a mean of 3.6 recruitment reminder calls were made to reach potential participants.^30^ If we cannot identify a person’s telephone number, we will send a reminder via post together with the invitation pack. Before either reminder type, we will receive up-to-date information concerning the living status of the person treated for cancer during childhood from the Swedish Tax Agency’s Population Registry via Uppsala University IT Services on the same day as sending the reminder to their parent. Those who do not consent by the time recruitment closes (ie, the sample size of 60 participants is reached or a recruitment period of 3 months has passed) are classified as lost to recruitment.

#### Recruitment timeline

We commenced recruitment in June 2026 and will recruit for a maximum of 3 months. Based on enrolment rates observed in the ENGAGE feasibility study and weekly invitation blocks of approximately 100 parents, we anticipate reaching a sufficient number of potentially eligible parents after sending approximately 6–7 blocks. Assuming a 9% randomisation rate, this would yield approximately 54–63 participants, sufficient to achieve the target sample of 60 participants, equivalent to approximately 20 participants per month over the 3-month recruitment period.

#### Consent

Those who wish to participate provide consent by signing the consent form and returning it to the research team using the freepost return envelope. Potential participants provide their telephone number and preferred times and days to be contacted by the research team when returning the consent form. A personal copy of the consent form can be retained.

### Randomisation: intervention or control group

After assessing eligibility during a structured inclusion interview via telephone (see Data collection methods), eligible participants complete the baseline assessment and are thereafter randomly allocated to the intervention or control group in a 1:1 ratio using block randomisation with a block size of 6. The randomisation sequence is computer-generated to ensure unpredictability and conducted through an online software application (https://www.sealedenvelope.com/) by an individual external to the research team. Following randomisation, the individual external to the research team will notify the research team of the participant’s allocation, after which a research team member will inform the participant of their allocation via SMS. For those allocated to the intervention group, a research team member will provide: (1) workbooks (via post together with an introduction note), (2) access to audio files and video case vignettes (online platform link via SMS), and (3) information about their assigned parent guide (via SMS). The research team will notify the assigned parent guide that they have been allocated a participant and the parent guide will contact the participant within 1 week to introduce themselves and schedule the initial assessment session.

### Blinding

Due to the nature of the intervention, participants and parent guides cannot be blinded to group allocation. We will not blind research team members who conduct data collection at post-treatment (12 weeks) and follow-up (6 months post-treatment) assessments. This decision is made because (1) this is a pilot RCT examining feasibility not designed to make inferential statements about potential between-group outcome differences, and (2) the primary outcomes are feasibility outcomes (eg, completion rates and missing data) which are objective and not subject to assessment bias. Blinded outcome assessment was considered but judged impractical given the small size of the research team, who combine trial coordination with data collection. To mitigate potential assessor bias, research team members collecting outcome data will follow a standardised, structured telephone assessment script to collect data, and all collected data are independently double-entered with discrepancies identified and resolved (see Data recording and entry). In a future superiority RCT, research team members conducting outcome assessments will be blinded to group allocation.

### Data collection methods

We will collect data during pre-enrolment, enrolment, and post-randomisation (see table 3).

#### Sociodemographic and medical data

##### Pre-enrolment: identification and invitation via registries

We will first request information from the Swedish Childhood Cancer Registry about persons treated for cancer during childhood: personal identification number to identify the person’s parents in the Swedish Tax Agency’s Population Registry; diagnostic information (age at diagnosis, date of diagnosis, diagnostic group (CSN tumours, leukaemias, lymphomas, solid tumours) and predisposing previous cancer); and treatment dates (end date of primary treatment, end date of relapse treatment and end date of latest treatment).

We will thereafter request information from the Swedish Tax Agency’s Population Registry. For persons treated for cancer during childhood: personal identification number; reason for deregistration from the registry including date of deregistration; mother’s personal identification number; father’s personal identification number; and guardian’s personal identification number, with date of custody. For parents: personal identification number; reason for deregistration from the registry including date of deregistration; name; public registration address; special mailing address; place of birth, place of birth abroad (if relevant); and country of birth.

##### Enrolment: inclusion interview and baseline assessments

On receiving a potential participant’s written consent, we will book, via SMS, a time for the inclusion interview. If the first SMS is not answered, we will send up to two reminders via SMS within 1 week. If these are not answered, we will make up to two attempts to contact the potential participant via telephone the following week. The number of reminder attempts is informed by ENGAGE procedures, where participants were reminded up to five times (telephone, SMS, e-mail) to complete assessments.^30^ Ahead of a booked time, we will send a confirmation and a reminder via SMS 7 days and two working days (if applicable), respectively, before a booked time.

We will assess eligibility during a structured inclusion interview via telephone. If a potential participant fulfils inclusion criteria, they are asked structured questions about their sociodemographic characteristics and medical data for the person treated for cancer during childhood (≈15 min). We will collect the following sociodemographic characteristics for study participants: date of birth, gender identity, relationship and cohabitation status, education level, number and age of children and health problems among other children, housing situation, country of birth and their parents’ country/countries of birth, native language, and previous psychological treatment. We will collect the following sociodemographic characteristics and medical data about the person treated for cancer during childhood: date of birth, gender identity, cancer diagnosis/es, time since first diagnosis, time since end of last treatment, type of cancer treatment/s, and cancer recurrence (yes/no). Thereafter, participants will directly proceed to the baseline assessment via telephone (≈30 min).

##### Post-randomisation: assessment at post-treatment and follow-up

Research team members will collect data via telephone (≈30 min) at post-treatment (12 weeks), and follow-up (6 months post-treatment). We will book a time for assessments via SMS or telephone following the same procedure as for the inclusion interview and baseline assessment.

##### Data recording and entry

Data collected via telephone will initially be recorded on paper forms and subsequently entered independently into Microsoft Excel by two research team members in accordance with standard operating procedures. Entries will be compared and any discrepancies will be identified and resolved.

### Procedures to promote participant retention

We will provide participants who complete the post-treatment (12 weeks) and follow-up (6 months post-treatment) assessments with a gift voucher (100 SEK, approximately €9) after each assessment.

### Outcome data collected for participants who withdraw from trial and/or intervention

All participants will be invited to complete post-treatment (12 weeks) and follow-up (6 months post-treatment) assessments according to the intention-to-treat principle. Participants who withdraw from the intervention but remain in the trial will continue to complete all planned assessments. Participants who withdraw from the trial will not be contacted for further assessments, data collected up to the point of withdrawal will be retained and included in analyses where appropriate.

### Data management

Research data (data) includes: personal data (ie, code list, consent forms, contact details, and data from the Swedish Childhood Cancer Registry and Swedish Tax Agency’s Population Registry); and pseudonymised data (ie, inclusion interviews, assessments at baseline, post-treatment (12 weeks), and follow-up (6 months post-treatment).

A data management plan using DMPonline will be prepared and all data will be processed in accordance with Swedish legislation and the EU General Data Protection Regulation (GDPR, EU 2016/679). We have conducted a data security assessment and information classification in accordance with the Swedish Civil Contingencies Agency guidelines and the principles of Confidentiality, Integrity, and Availability (CIA).

We will store data in fireproof cabinets at Uppsala University and/or in the secure storage solution Allvis, a web-based platform provided by Uppsala University for storing research data.

#### Personal data

We will store the code list (Excel file) linking the unique ID number and personal identification number of each participant in a storage area in Allvis protected by encryption with VeraCrypt, and the code list will be stored separately from other personal and pseudonymised data. All pseudonymised data can be traced back to participants via the code list with unique ID numbers and personal identification numbers.

We will store signed consent forms (paper based), which include participants’ names, personal identification numbers and telephone numbers, in a locked fireproof cabinet at Uppsala University, and signed consent forms will be stored separately from other personal and pseudonymised data.

We will store contact details (Excel file), which includes participants’ personal identification numbers, names, addresses and telephone numbers, in a storage area in Allvis, and contact details will be stored separately from other personal and pseudonymised data.

We will store data from the Swedish Childhood Cancer Registry (Excel file) on a password protected encrypted USB stick in a locked fireproof cabinet at Uppsala University, and data from the Swedish Childhood Cancer Registry will be stored separately from other personal and pseudonymised data.

We will store data from the Swedish Tax Agency’s Population Registry (Excel file) in a storage area in Allvis protected by encryption with VeraCrypt, and data from the Swedish Tax Agency’s Population Registry will be stored separately from other personal and pseudonymised data.

#### Pseudonymised data

We will store pseudonymised data from inclusion interviews, assessments at baseline, post-treatment (12 weeks) and follow-up (6 months post-treatment) (paper based and Excel files) together in a locked fireproof cabinet at Uppsala University and in a storage area in Allvis and, pseudonymised data will be stored separately from personal data.

We will follow Uppsala University guidelines regarding management, storage and archiving of data. We will store passwords for USBs and VeraCrypt passwords securely using a password manager. Only the principal investigator and authorised research team members have access to the code list and password manager.

We will store all data, including the code list, for a period of 10 years after the date of the last publication. At this date, a decision will be made by the principal investigator to either continue to store or destroy the data. If there is no longer a need to identify participants, for example, for data verification purposes, the code list will be destroyed. As the destruction of data is carried out at least 10 years after the date of the last publication, it is not possible to specify in advance exactly how data will be destroyed, however, this will take place in accordance with Uppsala University guidelines on data management, storage and archiving of data at that time.

### Statistical methods

Quantitative data analysis is primarily descriptive and addresses outcomes related to procedural, intervention, and methodological uncertainties. Research team members will conduct the analysis following a pre-specified statistical analysis plan, with PAB members contributing to sense-making and interpretation of results. We will illustrate trial flow using a CONSORT diagram for pilot and feasibility studies.^51^

We will use independent samples t-test (continuous data) and χ² test (categorical data) to examine potential differences in sociodemographic characteristics between those invited versus those who consent.

We will calculate the proportion of those invited who consent, are assessed for inclusion, are included and are randomised, alongside 95% CIs. We will calculate the proportion of participants in the intervention and control group who complete questionnaires at baseline, post-treatment (12 weeks), and follow-up (6 months post-treatment), alongside 95% CIs. We will calculate the proportion of responses missing in each questionnaire for the intervention and control group at baseline, post-treatment (12 weeks), and follow-up (6 months post-treatment), alongside 95% CIs. We will calculate the proportion of the intervention group who complete the minimum treatment dose, alongside 95% CIs.

For clinical, health economic and process outcomes, we will report means and standard deviations (SDs) for normally distributed continuous variables, medians and interquartile ranges (IQRs) for non-normally distributed continuous variables, and frequency and percentages for categorical variables.

We will calculate the SD around the mean score on mental well-being (QWB) for the intervention and control group at post-treatment (12 weeks) and follow-up (6 months post-treatment) to assess variance in mental well-being. We will calculate correlation (Spearman’s ρ) between baseline QWB scores and scores at post-treatment (12 weeks) and follow-up (6 months post-treatment). We will estimate differences in mental well-being between the intervention and control groups by calculating mean changes in the QWB from baseline to post-treatment (12 weeks) and from baseline to follow-up (6 months post-treatment), alongside 95% CIs.

#### Missing data

Given the focus on feasibility assessment, we will not impute missing data. Instead, we will report the amount of missing data to inform decisions regarding the handling of missing data in a future superiority RCT.

#### Decision-making about progression to a future superiority RCT

To inform the decision to progress to a future superiority RCT, we will consider the pre-specified progression criteria, feasibility outcomes without progression criteria and findings from the process evaluation.^53^ We will determine, in collaboration with the TSC, whether feasibility outcomes suggest GO, AMEND with modifications or STOP.

### Data monitoring committee

Given this is a pilot trial examining feasibility rather than effectiveness and considering the low-risk nature of the intervention, we consider that a formal independent data monitoring committee is not required. We have established a TSC, including independent researchers, to advise on any safety concerns that may arise during the trial (see Trial Monitoring).

#### Interim analyses and stopping guidelines

We have no planned interim analyses for efficacy or futility as this is a pilot trial not powered to evaluate effectiveness. We will monitor SAEs on an ongoing basis. If an SAE is reported, the principal investigator will review it immediately and consult the TSC if needed. The principal investigator has the authority to terminate the trial if serious safety concerns arise.

### Trial monitoring

#### Trial management group

A Trial Management Group (TMG), consisting of the principal investigator, research team members, one PAB coordinator and parent guides, will meet at least every 2 weeks from trial setup until trial completion. The TMG oversees trial set-up, ongoing trial management, data collection, analysis, interpretation of findings, and dissemination activities.

#### Trial steering committee

We have established a TSC to provide trial oversight, including monitoring progression against the agreed timeline, ensuring adherence to the protocol and data analysis plan, and advising on major procedural and methodological decisions. The TSC includes the principal investigator (or designated research team member), one additional research team member, three external researchers with relevant methodological and/or clinical experience, and two PAB members. The TSC will meet approximately monthly during the recruitment period and thereafter every 2 months until trial completion. The TSC will review recruitment and retention data, SAEs, and provide recommendations to the research team; however, final decision-making authority rests with the principal investigator.

## Ethics and dissemination

The CHANGE-pilot RCT was approved by the Swedish Ethical Review Authority on February 2, 2026 (Dnr 2025–09024-01) and is conducted in accordance with the Declaration of Helsinki.

All potential participants will provide written informed consent and will be informed of their rights to withdraw from the trial and/or the intervention without providing a reason. We developed information material in collaboration with the PAB. Invitation packs include contact details for the principal investigator, the external PAB coordinator (a parent with lived experience) and the Uppsala University Data Protection Officer, should potential participants and participants have any concerns regarding trial conduct. All research team members involved in data collection will be trained and supervised by researchers with experience in conducting similar trials. Research team members who have contact with potential participants and participants will be provided with training to ensure basic knowledge about childhood cancer, for example, diagnoses and treatments. No biological specimens are collected in this trial.

We will publish the results in peer-reviewed journals, present at national and international conferences, and disseminate to relevant organisations. We will co-produce, in collaboration with the PAB, a dissemination plan targeting outside of academia, for example, via cancer organisations, including strategies for local dissemination, and will co-develop plain language summaries for participants.

### Trial sponsor

Uppsala University, Box 256, 751 05 Uppsala, Sweden

### Data availability statement

Data sharing is not applicable to this manuscript as no data were created or analysed. We will deposit anonymised quantitative data collected in the public data repository Zenodo. Potential participants will be informed of the data-sharing plans in the participant information sheet and provide informed consent accordingly. Before sharing, we will remove all personal identifiers or codes to ensure individuals cannot be identified. We will register metadata in the Swedish National Data Service (SND) catalogue, in accordance with national recommendations from the Swedish Research Council (Vetenskapsrådet), to ensure discoverability through SwePub/SND.

## Supplementary material

10.1136/bmjopen-2026-124267 online supplemental table 1
