# Supplementary material for: A workbook- and audio-administered, telephone-guided, low-intensity cognitive behavioural therapy intervention (EJDeR) for parents of persons treated for cancer during childhood: protocol for the CHANGE-pilot randomised controlled trial
Source: BMJ Open. 2026 Sep 22;16(9):e124267. doi: 10.1136/bmjopen-2026-124267 (PMC13599942; doi:10.1136/bmjopen-2026-124267)
Supplement: Supplementary file 1 [file bmjopen-2026-124267.reviewer_comments.pdf]

PEER REVIEW HISTORY

BMJ Open publishes all reviews undertaken for accepted manuscripts. Reviewers are asked to complete a checklist review form and are provided with free text boxes to elaborate on their assessment. These free text comments are reproduced below.

ARTICLE DETAILS

Title (Provisional)

A workbook- and audio-administered, telephone-guided, low-intensity cognitive behavioural therapy intervention (EJDeR) for parents of persons treated for cancer during childhood: protocol for the CHANGE-pilot randomised controlled trial

Authors

Woodford, Joanne; Reuther, Christina; Asberg Dun, Louise; Farrand, Paul; Johansen, Kine; Board, Parent Advisory; Richards, David A; Sampaio, Filipa; Thiblin, Ella; Von Essen, Louise

---

VERSION 1 - REVIEW

**Reviewer**	**1**
**Name**	**Bradford, Natalie**
**Affiliation**	**Queensland University of Technology, Cancer and Palliative Care Outcomes Centre**
**Date**	**10-Aug-2026**
**COI**	**None**

This is a well-designed feasibility pilot RCT protocol addressing an important and under-served area — psychological support for parents of childhood cancer survivors after treatment completion. The use of a pre-specified GO-AMEND-STOP progression framework and a Parent Advisory Board embedded throughout the trial are notable strengths. However, the manuscript would benefit from revision before publication, primarily around: (1) clarity of the intervention's developmental history and terminology (ENGAGE/CHANGE/EJDeR), (2) missing methodological detail needed to judge feasibility (progression criteria thresholds, sample size rationale, fidelity assessment), and (3) some internal inconsistencies that need reconciling. I recommend major revisions.

-Major comments

1. Abstract — scope of psychological support gap. The abstract states that only a minority of parents have access to psychological support after treatment completion. Is support also lacking during treatment? If the rationale is a specific post-treatment gap, this should be stated explicitly; if psychological support is similarly limited during treatment, this is worth

acknowledging, as it affects how the case for a post-treatment-focused intervention is framed.

2. Intervention development history is unclear. The manuscript refers to at least three related labels — ENGAGE, CHANGE, and EJDeR — without making the relationship between them clear to the reader.

- The ENGAGE feasibility trial is described as single-arm; please report the number of participants enrolled and relevant proportions (e.g., completion, retention).

- The CHANGE Pilot RCT is introduced later (p7, line 12) without clarifying whether this is the same study as ENGAGE, a successor to it, or a separate study.

- The rationale for the CHANGE pilot RCT (p8, line 29) appears before the reader has a clear picture of how it differs from the prior pilot(s) or how it relates to the currently proposed EJDeR protocol.

- Suggestion: Add a clear timeline/diagram of the intervention's development (e.g., ENGAGE → CHANGE → EJDeR), and use section headings to mark each development cycle. This would substantially improve readability given the number of acronyms in use.

3. Reconcile "minimum treatment dose" statements. Page 10, line 7 lists "proportion completing the minimum treatment dose" as an outcome, yet elsewhere the manuscript states the minimum treatment dose is uncertain) p9, line 8 “Uncertainties exist regarding whether modifications impact completion of the minimum treatment dose and parent guide fidelity to the intervention.”

Please reconcile these two statements, either define the minimum dose being measured, or clarify that defining it is itself a feasibility objective of this trial.

4. Sample size rationale absent. No justification is provided for the target of 60 participants. While formal power calculations are not expected for pilot trials, a rationale (e.g., precision-based estimation of recruitment/retention rates, or established pilot-trial sample size guidance) should be included particularly given the prior work to develop the intervention and previous trials

5. Recruitment timeline and prior publication. A related protocol paper (ref 51) is also referenced, and the trial is described as already recruiting, with only 3 months of recruitment planned. Please clarify: the assumed eligible/recruitable population per month supporting recruitment of 60 participants in this windows.

Minor comments

6. Timing of outcome measures. Methods and analysis (line 47) describes assessment timing as "post-treatment (12 weeks)" and "follow-up (6-months post-treatment)." Given the paper is about parents of children treated for cancer, "post-treatment" risks being misread as referring to the child's cancer treatment. Suggest replacing with "post-intervention" throughout.

7. Keywords. Consider adding "child" and/or "childhood cancer" to the keyword list — the current list (Psychological Well-Being, Anxiety, Depression, Clinical Trial, Parents, Low-Intensity, Cognitive Behavioral Therapy, Feasibility Studies, Cancer Survivors) does not clearly signal the paediatric context on its own.

8. Assessor blinding. The authors appropriately note in the limitations that assessors are unblinded. It would strengthen the protocol to briefly state whether blinded assessment was considered and why it was not feasible, and whether any other bias-mitigation steps (e.g., standardised assessment scripts, independent data checks) are planned.

9. Safety/escalation protocol. Given the intervention targets depression, anxiety, and PTSD symptoms in a potentially vulnerable population, please describe what safety or escalation procedures are in place for participants who deteriorate or disclose risk during the trial.

10. Intervention fidelity. Please clarify whether and how intervention fidelity is assessed (e.g., session logs, recorded calls, adherence checklists), as this is standard for behavioural intervention feasibility trials and directly informs the "intervention uncertainty" component of the progression framework.

11. Language/generalisability. The Swedish-language-only limitation is appropriately noted. Given Sweden's linguistically diverse population and that language/cultural barriers may compound unmet psychological need in families facing childhood cancer, it would be worth briefly noting whether translated or culturally adapted materials were considered for a future full trial.

VERSION 1 - AUTHOR RESPONSE

Reviewer Comments to Author:

<u>Reviewer: 1</u>
Comments to the Author: This is a well-designed feasibility pilot RCT protocol addressing an important and under-served area — psychological support for parents of childhood cancer survivors after treatment completion. The use of a pre-specified GO-AMEND-STOP progression framework and a Parent Advisory Board embedded throughout the trial are notable strengths.
Thank you for this comment.

However, the manuscript would benefit from revision before publication, primarily around: (1) clarity of the intervention's developmental history and terminology (ENGAGE/CHANGE/EJDeR), (2) missing methodological detail needed to judge feasibility (progression criteria thresholds, sample size rationale, fidelity assessment), and (3) some internal inconsistencies that need reconciling. I recommend major revisions:
Thank you for identifying areas where clarity could be improved. We address each comment below.

1. Abstract — scope of psychological support gap. The abstract states that only a minority of parents have access to psychological support after treatment completion. Is support also lacking during treatment? If the rationale is a specific post-treatment gap, this should be stated explicitly; if psychological support is similarly limited during treatment, this is worth acknowledging, as it affects how the case for a post-treatment-focused intervention is framed.

Our rationale concerns a specific post-treatment gap. Parents have described the transition from hospital-based care to the post-treatment period as particularly challenging, as healthcare is abruptly withdrawn following treatment completion, and some parents report not feeling prepared for this transition. We have revised the manuscript to state this explicitly.

> Abstract, page 2:
> In Sweden psychosocial support is available to parents via paediatric oncology care during cancer treatment, however only a minority of parents have access to psychological support after treatment completion.
>
> Introduction, page 4:
> Parents have described the transition from hospital care to the time after cancer treatment as particularly challenging, as healthcare is abruptly withdrawn, with some parents reporting being inadequately prepared for the post-treatment period.[2] Whilst psychosocial support is available to parents via paediatric oncology care during cancer treatment[16], only a minority of parents in Sweden receive psychological support after treatment completion, and access declines over time.[17]

2. Intervention development history is unclear. The manuscript refers to at least three related labels — ENGAGE, CHANGE, and EJDeR — without making the relationship between them clear to the reader.
- The ENGAGE feasibility trial is described as single-arm; please report the number of participants enrolled and relevant proportions (e.g., completion, retention).
Thank you for this comment. We have now provided more detail concerning the results of the ENGAGE feasibility trial:

> Introduction, page 5:
> We enrolled 75 participants (56 identified via the Swedish Childhood Cancer Registry and Swedish Tax Agency's Population Registry, and a further 19 via other recruitment strategies), of which 72 completed baseline assessments and gained access to EJDeR.
> Five progression criteria met GO: (1) 11.0% (56/509) were enrolled of total potential participant population invited via the Swedish Childhood Cancer Registry and Swedish Tax Agency's Population Registry, criteria ≥9.0%; (2) 24% (18/75) dropped out of the trial and 23.6% (17/72) dropped out of the intervention, criteria ≤30.0%; (3) 0.01–3.9% of questionnaire items were missing, criteria ≤10.0%; (4) 98.7% (74/75), 84.4% (54/64), and 81.4% (48/59) completed telephone interviews at baseline, post-treatment (12 weeks), and follow-up (6-months post-treatment) respectively of those remaining in the study at each time-point, criteria ≥70.0%; and (5) no substantial negative consequences were reported. Two criteria met AMEND: (1) 65.6% (42/64) and 67.8% (40/59) completed online assessments at post-treatment (12 weeks) and follow-up (6-months post-treatment) respectively of those remaining in the study at each time-point, criterion ≥70.0%); and (2) 47.9% (34/71) completed the minimum treatment dose completion, criterion ≥50.0%.

- The CHANGE Pilot RCT is introduced later (p7, line 12) without clarifying whether this is the same study as ENGAGE, a successor to it, or a separate study.
- The rationale for the CHANGE pilot RCT (p8, line 29) appears before the reader has a clear picture of how it differs from the prior pilot(s) or how it relates to the currently proposed EJDeR protocol.
- Suggestion: Add a clear timeline/diagram of the intervention's development (e.g., ENGAGE → CHANGE → EJDeR), and use section headings to mark each development cycle. This would substantially improve readability given the number of acronyms in use.
Thank you for this comment. We agree the relationship between EJDeR, ENGAGE, and the CHANGE-pilot RCT required clarification and have made a number of changes.

(1) We have made small modifications to the order of the Introduction section.

(2) We have added a Figure to explain the timeline (see Figure 1, page 7)
(3) We have made the following modifications to the text:

Introduction, page 4:
Given the aforementioned unmet support needs of parents of persons treated for cancer during childhood, we conducted iterative intervention development and feasibility research (Figure 1), following the Medical Research Council (MRC) framework.[18]

Introduction, page 5:
Research from the intervention development phase informed the feasibility phase (Figure 1) where we conducted the single-arm feasibility trial ENGAGE[30] to examine procedural, intervention, and methodological uncertainties around the internet-administered version of EJDeR and planned trial procedures for a future evaluation of EJDeR in a superiority randomised controlled trial (RCT). Progression criteria were set a priori, following a GO-AMEND-STOP progression framework.[31]

Introduction, page 6:
Therefore, we progress via an external pilot RCT with progression criteria set[31] (the CHANGE-pilot RCT, Figure 1) to examine remaining procedural, intervention, and methodological uncertainties around the workbook- and audio-administered version of EJDeR and planned trial procedures. Findings will inform the design and conduct of a future full-scale evaluation of EJDeR in a superiority RCT of EJDeR plus usual care (intervention group) versus usual care alone (control group).

3. Reconcile "minimum treatment dose" statements. Page 10, line 7 lists "proportion completing the minimum treatment dose" as an outcome, yet elsewhere the manuscript states the minimum treatment dose is uncertain) p9, line 8 "Uncertainties exist regarding whether modifications impact completion of the minimum treatment dose and parent guide fidelity to the intervention."
Please reconcile these two statements, either define the minimum dose being measured, or clarify that defining it is itself a feasibility objective of this trial.
Thank you for this comment. We have clarified that the minimum treatment dose (MTD) is defined a priori (attendance at the initial assessment session and completion of at least four weekly telephone guidance sessions; see Minimum treatment dose). What is uncertain, and is examined as an intervention uncertainty, is not the definition of the MTD itself but whether parents can complete the MTD given the modifications made to EJDeR since the ENGAGE feasibility study.

Introduction, page 8:
Since conducting ENGAGE, we modified EJDeR with respect to content, mode of delivery, and type of guidance and defined a revised minimum treatment dose (see Minimum treatment dose). Uncertainties exist regarding the proportion of participants who will complete the minimum treatment dose when receiving the modified intervention, and regarding parent guide fidelity to the EJDeR guidance protocols. We will therefore examine these intervention uncertainties to ensure the intervention is feasible to participants and parent guides.

4. Sample size rationale absent. No justification is provided for the target of 60 participants. While formal power calculations are not expected for pilot trials, a rationale (e.g., precision-based estimation of recruitment/retention rates, or established pilot-trial sample size guidance) should be included particularly given the prior work to develop the intervention and previous trials
We have provided more detail to the sample size rationale:

Method and analyses, page 22:
We will recruit 60 participants (30 to the intervention group, 30 to the control group), consistent with the median sample size per arm reported for pilot trials.[76] With 30 participants per group

we can estimate proportions with reasonable precision: 9.0% randomisation rate (approximately 60/667 invited; 95% CI 7.0%–11.4%), 70.0% completion of all questionnaires at baseline, post-treatment (12 weeks), and follow-up (6-months post-treatment) (42/60; 95% CI 57.4%–80.2%), and completion of the minimum treatment dose in at least 65% of participants in the intervention group (20/30; 95% CI 46.0%–83.6%). These estimates will provide adequate information to inform progression decisions.

5. Recruitment timeline and prior publication. A related protocol paper (ref 51) is also referenced, and the trial is described as already recruiting, with only 3 months of recruitment planned. Please clarify: the assumed eligible/recruitable population per month supporting recruitment of 60 participants in this window.
We have added the following text which we hope clarifies:

> Method and analyses, page 23:
> We commenced recruitment in June 2026 and will recruit for a maximum of three months. Based on enrolment rates observed in the ENGAGE feasibility study and weekly invitation blocks of approximately 100 parents, we anticipate reaching a sufficient number of potentially eligible parents after sending approximately 6–7 blocks. Assuming a 9% randomization rate, this would yield approximately 54–63 participants, sufficient to achieve the target sample of 60 participants, equivalent to approximately 20 participants per month over the three month recruitment period.

6. Timing of outcome measures. Methods and analysis (line 47) describes assessment timing as "post-treatment (12 weeks)" and "follow-up (6-months post-treatment)." Given the paper is about parents of children treated for cancer, "post-treatment" risks being misread as referring to the child's cancer treatment. Suggest replacing with "post-intervention" throughout.
Thank you for this comment. We appreciate the concern regarding potential ambiguity. However, “post-treatment” is the term used throughout our published programme of work on this intervention (e.g., the ENGAGE feasibility study and related publications) and we have chosen to retain it here for consistency across this wider body of research, rather than introduce a new term partway through a multi-study programme.

7. Keywords. Consider adding "child" and/or "childhood cancer" to the keyword list — the current list (Psychological Well-Being, Anxiety, Depression, Clinical Trial, Parents, Low-Intensity, Cognitive Behavioral Therapy, Feasibility Studies, Cancer Survivors) does not clearly signal the paediatric context on its own.
Thank you for this comment. We have added “Child” and “Childhood Cancer” to the keyword list.

> Keywords, page 3:
> “Psychological Well-Being, Anxiety, Depression, Clinical Trial, Parents, Child, Childhood Cancer, Low-Intensity, Cognitive Behavioral Therapy, Feasibility Studies, Cancer Survivors”

8. Assessor blinding. The authors appropriately note in the limitations that assessors are unblinded. It would strengthen the protocol to briefly state whether blinded assessment was considered and why it was not feasible, and whether any other bias-mitigation steps (e.g., standardised assessment scripts, independent data checks) are planned.
Thank you for this comment. We have revised the manuscript to explain that blinded outcome assessment was considered but judged impractical given the small research team, and to detail the bias-mitigation steps already planned.

> Method and analyses, page 24:
> Blinded outcome assessment was considered but judged impractical given the small size of the research team, who combine trial coordination with data collection. To mitigate potential assessor bias, research team members collecting outcome data will follow a standardised,

structured telephone assessment script to collect data, and all collected data are independently double-entered with discrepancies identified and resolved (see Data recording and entry).

9. Safety/escalation protocol. Given the intervention targets depression, anxiety, and PTSD symptoms in a potentially vulnerable population, please describe what safety or escalation procedures are in place for participants who deteriorate or disclose risk during the trial.
Thank you for this comment. We have expanded the Harms section to describe the escalation procedure in more detail. Research team members conducting telephone assessments and parent guides providing telephone guidance are trained to identify risk during calls.

> Method and analyses, page 12:
> Procedures for identifying and responding to participant deterioration or disclosure of risk during the trial are described under Harms.
>
> Method and analyses, page 17-18:
> A licensed psychologist will train research team members working with recruitment and data collection to identify signs of serious mental illness and/or risk of suicide. In acute situations, e.g., where a participant discloses an immediate risk to their safety, research team members will contact emergency services regardless of the participant's response. In non-acute situations, e.g., suicidal ideation without immediate risk, we will inform the participant that we would like a licensed psychologist in our research team to contact them, and, if the parent gives permission, the licensed psychologist will contact them within two working days to conduct a risk assessment. If the participant does not give permission, they are encouraged to contact primary care. If a parent is assessed as needing more support than we can provide within the trial, we will direct them to appropriate mental health services and exclude them from the trial. If a parent guide identifies signs of serious mental illness (including deterioration) and/or risk of suicide, e.g., when receiving telephone guidance from the parent guide, the parent guide will conduct the risk assessment immediately and consult with the parent guide supervisor if needed.

10. Intervention fidelity. Please clarify whether and how intervention fidelity is assessed (e.g., session logs, recorded calls, adherence checklists), as this is standard for behavioural intervention feasibility trials and directly informs the "intervention uncertainty" component of the progression framework.
Thank you for this comment. Intervention fidelity assessment is described in detail in the separate protocol for the embedded mixed methods process evaluation referenced in this manuscript (reference 53). We have added the following text to make this explicit, and briefly summarised the approach.

> Method and analyses, page 14:
> Sessions will be audio recorded (with consent) and we will select a random 15% of recorded sessions to examine parent guide fidelity to the EJDeR guidance protocols. The sampling strategy and fidelity assessment method are described in full in the process evaluation protocol.[53]

11. Language/generalisability. The Swedish-language-only limitation is appropriately noted. Given Sweden's linguistically diverse population and that language/cultural barriers may compound unmet psychological need in families facing childhood cancer, it would be worth briefly noting whether translated or culturally adapted materials were considered for a future full trial.
Thank you for this comment. We agree this is an important consideration. We have discussed when cultural adaptation should take place and, given the time and resources required to undertake this appropriately in collaboration with parents from different linguistic groups in Sweden, we decided to first assess the feasibility of EJDeR in Swedish. We plan to seek funding to subsequently culturally adapt the intervention for other groups, including ethnic minority populations.

In the current study, we also collect sociodemographic characteristics relevant to assessing representativeness, including country of birth and native language. This data will enable us to examine differences between consenting and declining parents, and thus identify groups that may be underrepresented, and inform priorities for future cultural adaptation to ensure the intervention is feasible and acceptable to diverse groups of parents.

We have added the following text.

> Strengths and limitations of the study, page 3:
> The study is limited to Swedish-speaking parents living in Sweden, which limits generalisability to other settings and populations. Future research should include cultural adaptation of the intervention to improve its relevance for linguistically and culturally diverse parents.

VERSION 2 - REVIEW

Reviewer **1**

Name **Bradford, Natalie**

Affiliation **Queensland University of Technology, Cancer and Palliative Care Outcomes Centre**

Date **07-Sep-2026**

COI

Thank you for comprehensively addressing the queries raised. I am satisfied that all are now addressed, and the protocol is suitable for publication. Good luck with this research!
