# Supplementary material for: A workbook- and audio-administered, telephone-guided, low-intensity cognitive behavioural therapy intervention (EJDeR) for parents of persons treated for cancer during childhood: protocol for the CHANGE-pilot randomised controlled trial
Source: BMJ Open. 2026 Sep 22;16(9):e124267. doi: 10.1136/bmjopen-2026-124267 (PMC13599942; doi:10.1136/bmjopen-2026-124267)
Supplement: Supplementary file 2 [file bmjopen-16-9-s001.docx]

**Supplemental table**

Table S1. Modifications to EJDeR content, delivery, and guidance informed by ENGAGE findings, ENGAGE public contributors, and the CHANGE-pilot RCT Parent Advisory Board.

|  | Rationale | Modification |
| --- | --- | --- |
| Content | ENGAGE participants^1^ and ENGAGE public contributors wanted further tailoring to the population, e.g., acknowledging cancer-related experiences, such as worries about late effects, and support needs for the child. | Content further tailored to the population, e.g., including more cancer-related examples in exercises. |
|  | ENGAGE participants appreciated the video case vignettes (i.e., case stories of parents who have used the intervention)^1^ and ENGAGE public contributors wanted video case vignettes that were more tailored to the population and adopted a more recovery-focused tone. | Additional video case vignettes added, focused on parents’ recovery stories, i.e., how working with EJDeR had helped them. |
|  | Adherence to the EJDeR minimum treatment dose (47.9%) met the ’AMEND’ criterion for progression (criterion ≥50%).^2^  Adherence to the worry management module, especially for fathers, was low,^2,3^ and fathers reported using more avoidant coping strategies than mothers.^3^ | Psychoeducation on motivation added and existing motivational exercises enhanced in the Introduction and Psychoeducation Module.  The rationale for worry management presented in the Worry Management Module adapted to include more information on the role of avoidance in maintaining anxiety. |
|  | ENGAGE public contributors suggested a need for a more positive tone.^4^ CHANGE-pilot RCT Parent Advisory Board suggested EJDeR can help with mental well-being, in addition to alleviating symptoms of depression and anxiety.^5^ | Language revised to emphasise mental well-being. Diagnosis-specific language, such as “depression”, removed throughout the intervention in favour of terms such as “low mood” or “feeling down, low, and tired”. |
|  | ENGAGE participants^1^ and ENGAGE public contributors wanted trauma acknowledged in EJDeR.  ENGAGE e-therapists^6^ reported challenges in supporting parents dealing with trauma-related difficulties. | Additional Psychoeducation and Resources Module added, including psychoeducation on common reactions to traumatic events.  Imagination exercises added to help with emotion regulation. |
|  | ENGAGE participants reported parenting, relationship, and sleep difficulties.^1,7^ | Additional Psychoeducation and Resources Module added, including psychoeducation on parenting, relationships, and sleep, as well as signposting to relevant resources for parents of persons treated for cancer during childhood, e.g., family counselling and financial counselling. |
| Delivery | ENGAGE participants^1^ and e-therapists^6^ reported challenges navigating the U-CARE Portal^8^, for example difficulties finding completed exercises and parent guide feedback, lack of smartphone and tablet compatibility, and challenges providing parents access to modules and adding homework exercises. PAB advised that providing EJDeR in audio form could improve adherence.^5^ | Mode of delivery adapted by providing a workbook- and audio-administered version of EJDeR. |
| Support and guidance | ENGAGE participants^1^ and e-therapists^6^ wanted more person-to-person communication via telephone or video-conferencing versus written messages. | Guidance adapted by providing weekly telephone guidance sessions with a parent guide. |
| *The U-CARE Portal is a web-based platform developed at Uppsala University, designed to deliver internet-administered psychological support and support digital research activities, including online consent and data collection. | | |

# REFERENCES

[1]	Thiblin E, Lundgren J, Bergqvist M, et al. The acceptability and feasibility of an internet-administered, guided, low-intensity cognitive behavioural therapy intervention for parents of children treated for cancer: findings from a qualitative study involving public contributors. BMC Psychiatry. 2025;25(1):499. doi: 10.1186/s12888-025-06897-y.

[2]	Thiblin E, Woodford J, Reuther C, et al. Internet-administered, low-intensity cognitive behavioural therapy for parents of children treated for cancer: a feasibility trial (ENGAGE). Cancer Med. 2023;12(5):6225-6243. doi: 10.1002/cam4.5377.

[3]	Lundgren J, Reuther C, Farrand P, et al. "It is today that counts, and today everything is fine": coping strategies utilized by parents of children treated for cancer who seek psychological support - a qualitative study. BMC Psychol. 2025;13(1):565. doi: 10.1186/s40359-025-02860-4.

[4]	Thiblin E, Woodford J, Berman AH, et al. Development and description of a logic model for a guided, low-intensity cognitive behavioural therapy intervention for parents of children treated for cancer: the EJDeR intervention. [Submitted to BMJ Open].

[5]	Reuther C, Thiblin E, Sugg HVR, et al. Involving parents of children treated for cancer as public contributors to inform the design of a trial of a low-intensity cognitive behavioural therapy self-help intervention: acceptability, feasibility, and impact. [Manuscript in preparation].

[6]	Reuther C, Lundgren J, Gottvall M, et al. E-therapists’ views on the acceptability and feasibility of an internet-administered, guided, low-intensity cognitive behavioural therapy intervention for parents of children treated for cancer: a qualitative study. Digit Health. 2024;10:20552076241260513. doi: 10.1177/20552076241260513.

[7]	Lundgren J, Thiblin E, Lutvica N, et al. Concerns experienced by parents of children treated for cancer: a qualitative study to inform adaptations to an internet-administered, low-intensity cognitive behavioural therapy intervention. Psychooncology. 2023;32(2):237-246. doi: 10.1002/pon.6074.

[8]	Sjöström J, Mustafa MI, Woodford J, et al. Designing software for web-based psychosocial care: longitudinal design science research in U-CARE. Commun Assoc Inf Syst. 2024;54:1153-1192. doi: 10.17705/1cais.05448.
